# Psychometric properties of the Depression Anxiety Stress Scales (DASS-21) in women with breast cancer

**DOI:** 10.1038/s41598-024-68814-9

**Published:** 2024-09-03

**Authors:** Lorena M. Soria-Reyes, Rafael Alarcón, María V. Cerezo, María J. Blanca

**Affiliations:** Department of Psychobiology and Behavioral Sciences Methodology, Faculty of Psychology and Speech Therapy, University of Málaga, Doctor Ortiz Ramos, 12, 29010 Málaga, Spain

**Keywords:** Distress, DASS-21, Validity evidence, Reliability, Breast cancer, Breast cancer, Quality of life, Human behaviour

## Abstract

Breast cancer impacts the psychological well-being of women, leaving them at risk of developing depression, anxiety, and other stress-related disorders. The Depression Anxiety Stress Scales (DASS-21) is a widely used measure, although empirical evidence regarding its psychometric properties in the breast cancer population is limited. The purpose of this study was to conduct an exhaustive analysis of the psychometric properties of the DASS-21 in a sample of Spanish women diagnosed with breast cancer. Participants were 289 breast cancer patients who completed the DASS-21 and other questionnaires measuring life satisfaction, positive and negative affect, flourishing, perceived stress, and breast cancer-specific stressors. In terms of validity evidence based on the internal structure of the DASS-21, adequate fit indices were obtained for the model based on three first-order factors (depression, anxiety, stress) and one second-order factor (general psychological distress). Reliability coefficients (McDonald’s omega) ranged from .84 to .95. Validity evidence based on relationships with other variables was also provided by moderate and strong correlations with well-being indicators and stress measures. The results support the use of the DASS-21 for measuring general psychological distress in the breast cancer context, where it may provide useful information for the design of psychological interventions with patients.

## Introduction

The incidence of cancer continues to grow, with estimates for our country, Spain, predicting as many as 286,664 new cases in 2024. Breast cancer is the most frequently diagnosed cancer among Spanish women, with an estimated total of 35,001 new cases in 2023^1^. Thanks to research, more advanced treatments, and primary screening, the overall 5-year survival rate for breast cancer in women is 90%^2^.

A diagnosis of breast cancer impacts the psychological well-being of women^3–5^, leaving them at risk of developing depression, anxiety, and other stress-related disorders^6^, which importantly may persist beyond the end of medical treatment^7^. The disease therefore needs to be addressed from a psycho-oncological perspective^8^.

Reliable and valid measurement tools are essential for assessing health indicators in the context of breast cancer. Widely used measures of psychological health include: for depression, the Beck Depression Inventory-II^9^ (BDI-II) and the Hamilton Depression Rating Scale^10^ (HDRS); for anxiety, the State-Trait Anxiety Questionnaire^11^ (STAI) and the Beck Anxiety Inventory^12^ (BAI); and for stress, the Perceived Stress Scale^13^ (PSS-14) and its shortened 10-item version^13,14^ (PSS-10), as well as specific stress measures developed specifically for the oncological context, such as the Questionnaire on Stress in Cancer Patients^15^ (QSC-R23), the Newly Diagnosed Breast Cancer Stress Scale^16^, and the Stressors in Breast Cancer Scale^17^ (SBCS). There are also instruments that assess depression and anxiety together, such as the Hospital Anxiety and Depression Scale^18^ (HADS).

However, because depression, anxiety, and stress present high comorbidity, Lovibond and Lovibond^19,20^ developed the Depression Anxiety Stress Scales (DASS), a composite measure of these negative emotional states. The DASS consists of 42 items distributed equally across the three scales (14 per scale), although the authors also developed a brief 21-item version^20^ (DASS-21) with 7 items per scale, which has shown similar psychometric properties to the original instrument^21^. Lovibond and Lovibond^19^ consider that the Depression scale evaluates negative emotional states such as dysphoria, hopelessness, devaluation of life, feeling gloomy or blue, being convinced that life has no meaning or value, pessimism about the future, and an inability to experience pleasure or satisfaction. The Anxiety scale assesses negative emotional states such as autonomic arousal, apprehension, sense of panic, situational anxiety, and the subjective experience of anxious affect. Finally, the Stress scale evaluates non-specific and chronic over-arousal, such as difficulty relaxing, nervous excitement, and ease of becoming angry/agitated, irritated, upset or impatient. Theoretically, stress can be understood as a stimulus or as a response^22,23^, with the Stress scale of the DASS-21 reflecting the idea of stress as a response.

The DASS-21 is widely used by psychologists to assess distress as it is brief, its items are easily understood by patients, and interpretation of responses is straightforward: high scores on each scale indicate higher levels of depression, anxiety or stress, while the total score is an indicator of overall distress. High scores on any of the scales would suggest the need for more in-depth assessment and, potentially, psychological treatment. Note too that the authors^20^ of the DASS-21 have established cut-off points for determining emotional states for each scale, and this can likewise help in psychological diagnosis. The tool is also useful for follow-up, as it can be re-administered to monitor progress and the effectiveness of intervention. Finally, the DASS-21 has exhibited better psychometric properties in breast cancer patients than have other similar tools, such as the HADS or the BDI-II^24^. It should be noted here that the tool has so far been translated into 55 languages, and hence its psychometric properties have been investigated worldwide^21,24–47^. In Spain, its psychometric properties have been analyzed by Ruiz et al.^25^, Daza et al.^30^, and Fonseca-Pedrero et al^31^.

Regarding validity evidence based on the internal structure of the DASS-21, two factorial structures have frequently been reported: (1) a model involving three correlated factors (depression, anxiety, and stress) based on the original scale^20,21,25,27,29,33,34,37–39,43–46^, and (2) a model proposed by Henry and Crawford^36^ comprising three first-order factors (depression, anxiety, and stress) and one second-order factor (general psychological distress)^21,30–32,36,40,41^. Both structures have been shown to be invariant across gender^27,33,37–40,46^, age^40,46^, countries^25,41^, time^28,44^, or group characteristics^25,33,42^.

Regarding reliability of DASS-21 scores, studies have reported internal consistency coefficients ranging from 0.80^31^ to 0.99^34^ for depression, 0.70^42^–0.99^34^ for anxiety, 0.71^25,26,31,45,46^–0.99^34^ for stress, and 0.74^35^–0.96^33^ for general psychological distress.

With respect to validity evidence based on relationships with other variables, scores on depression, anxiety, and stress, as well as the total DASS-21 score (general psychological distress), have shown convergent validity in different populations with scores on other instruments that also measure these constructs. Overall, as expected, scores on the DASS-21 are positively correlated with depression^26,29,30,32,34,35,42,45^, anxiety^26,29,30,32,34,45^, and stress^28^. Research has also shown that scores on the DASS-21 correlate positively with negative affect^36,38–40,46^ and negatively with several indicators of well-being^32,38,39^, specifically, life satisfaction^39^ and flourishing^42^, meaning in life^38,39^, resilience^38,39^, hope^38,39^, optimism^39^, gratitude^38,39^, and quality of life^38,42^. The relationship between DASS-21 scores and aspects related to mental health^32,33,37,38,42,43^ and suicidal ideation has likewise been examined^42^.

Although the DASS-21 has been used in the oncology population, there is limited empirical evidence regarding its psychometric properties^34,42,48^. Fox^42^ and Clover^48^ focused on different types of cancer patients and did not analyze the whole scale (only the Anxiety scale was used in the first of these studies, while the second used the Depression and Anxiety scales). Kumar^34^ administered the DASS-21 to a sample of patients with head and neck cancer or oral malignant disorders and conducted a principal component analysis with varimax rotation in which they specified four factors. Therefore, to the best of our knowledge, there are no psychometric studies of the DASS-21 in breast cancer patients. Consequently, the aim of this study was to conduct an exhaustive analysis of the psychometric properties of the DASS-21 in a sample of Spanish women diagnosed with breast cancer, a population that has yet to be studied. Specifically, we sought to provide validity evidence based on the internal structure, to carry out reliability and item analysis, and to obtain validity evidence based on relationships with other variables, including well-being indicators (life satisfaction, flourishing, and positive and negative affect) and measures of stress, both globally and cancer-specific. Based on previous evidence, we expected to find that DASS-21 scores (depression, anxiety, stress, and general psychological distress) are negatively related to scores on well-being indicators and positively associated with scores on negative affect and stress measures. Knowledge about the psychometric properties of the DASS-21 in the breast cancer context is important, given that inferences from an instrument should be made for a specific use, context, and population^49^.

## Method

### Sample

Participants were 289 Spanish women diagnosed with breast cancer and aged between 28 and 76 years (*M* = 51.09, *SD* = 8.76). They had a mean time since diagnosis of 4.70 years (*SD* = 5.41) and were recruited through different health centers providing cancer care in Spain. The inclusion criteria were a diagnosis of breast cancer, age 18 years or older, and signing informed consent. At the time of the study, 74.4% were married or living with their partner, 49.8% had completed high school and 41.5% university education, 32.5% were in employment, and 45% were at stage II of the TNM tumor classification system. Table 1 shows the sample characteristics.

**Table 1:** Sample characteristics (N = 289).

Variables	Percentage
Civil status	
Single/divorced/widowed	25.6
Married/living with partner	74.4
Educational level	
Elementary	8.7
High school	49.8
University	41.5
Employment status	
Working at home	17.0
Medical leave	23.5
Retired	18.3
Unemployed	8.7
Employed	32.5
Time since diagnosis (years)	
< 2	42.6
2–5	29.0
> 5	28.4
Cancer stage	
0	5.9
I	20.0
II	45.0
III	24.6
IV	4.5
Affected breast	
Left	46.4
Right	48.8
Both	4.8
Type of breast surgery	
Conservative	50.9
Mastectomy without reconstruction	23.5
Mastectomy with immediate reconstruction	11.8
Delayed reconstruction	9.0
No surgery	4.8

For the analysis of validity evidence based on relationships with other variables, the total sample was split randomly into two sub-samples (approximately 50–50) in order to administer different questionnaires and avoid fatigue among participants. Thus, in addition to the DASS-21 (all participants), the first sub-sample completed the Satisfaction with Life Scale (SWLS), Flourishing Scale (FS), and Positive and Negative Affect Schedule (PANAS), while the second subsample completed the Perceived Stress Scale (PSS-10) and the Stressors in Breast Cancer Scale (SBCS).

### Measures

*Depression Anxiety Stress Scales* (DASS-21)^20^, in its Spanish version^30^. The DASS-21 assesses feelings of depression, anxiety, and stress. It consists of three scales, each with seven items, and respondents are asked to indicate the extent to which the item statement has applied to them during the last week. Responses are given using a 4-point Likert-type scale (0 = does not apply to me at all; 3 = applies to me a lot or most of the time), and thus the total score for each scale ranges from 0 to 21, with high scores indicating a higher level of the respective construct. The total score for the DASS-21 (range from 0 to 63 points) is obtained by summing the total scores obtained on each of the three scales, with high scores reflecting a higher level of general psychological distress. Regarding the proposed cut-off points^20^ that can be used in clinical practice to determine emotional states for each scale, these are as follows: for the Depression scale, 0–9: normal, 10–13: mild, 14–20: moderate, 21–27: severe, and ≥ 28: extremely severe; for the Anxiety scale, 0–7: normal, 8–9: mild, 10–14: moderate, 15–19: severe, and ≥ 20: extremely severe; for the Stress scale, 0–14: normal, 15–18: mild, 19–25: moderate, 26–33: severe, and ≥ 34: extremely severe. The reliability analysis of scores obtained with the Spanish version of the DASS-21^30^ reported satisfactory results for the three scales and the total score (Cronbach's alpha coefficients > 0.85).

*Satisfaction with Life Scale* (SWLS)^50^, in its Spanish version^51^, which has also been validated with Spanish breast cancer patients^5^. The SWLS is a brief 5-item instrument for assessing life satisfaction as a whole. Each item is rated on a 7-point Likert-type scale (1 = strongly disagree; 7 = strongly agree), and high scores indicate a higher level of life satisfaction. McDonald’s omega coefficient in the present sample was 0.85.

*Flourishing Scale* (FS)^52^, in its Spanish version^53^. This 8-item scale assesses psychological well-being from the perspective of fullness in life. Each item is rated using a 7-point Likert-type scale (1 = strongly disagree; 7 = strongly agree). High scores correspond to a higher level of psychological growth or development. McDonald’s omega coefficient in the present sample was 0.82.

*Positive and Negative Affect Schedule* (PANAS)^54^, in its Spanish version^55^. The PANAS comprises 20 items describing emotions, divided into two subscales: Positive Affect (PA) and Negative Affect (NA), each with 10 items. Responses to each item are given using a 5-point Likert-type scale (1 = not at all; 5 = a lot). High scores on a given subscale suggest a stronger presence of the corresponding emotions. McDonald's omega coefficient in the present sample was 0.72 for positive affect and 0.76 for negative affect.

*Perceived Stress Scale* (PSS-10)^13,14^, in its Spanish version^56^, which has also been validated with Spanish breast cancer patients^57^. This scale comprises 10 items, each rated on a 5-point Likert-type scale (0 = never; 4 = very often), that measure the degree to which daily events are perceived as uncontrollable, unpredictable, and stressful. The PSS-10 yields scores on two factors: perceived helplessness, consisting of six items that assess perceived stress; and perceived self-efficacy, comprising four items referring to the perceived degree of coping ability with respect to current stressors. McDonald's omega coefficients in the present sample were 0.88 and 0.75, respectively.

*Stressors in Breast Cancer Scale* (SBCS)^17^, which consists of 24 items assessing breast cancer-specific stressors. The scale is divided into five subscales: physical appearance and sex strains, health and daily difficulties, interpersonal relationship strains, healthcare strains, and worries and concerns about the future. Each item is rated on a 5-point Likert-type scale (1 = not at all stressful or is irrelevant to me; 5 = very stressful), and a total score can be computed by summing the subscale scores. High scores indicate high levels of stress on the respective subscale. McDonald’s omega coefficient in the present sample was 0.94 for the total score, and ranged from 0.81 to 0.87 for scores on the five subscales.

### Procedure

The study complied with the ethical standards of the Declaration of Helsinki and was approved by the Experimental Ethics Committee of the University of Málaga. Participants were all volunteers and did not receive any incentives. The research team contacted by email and telephone the staff of different health centers providing care for women with breast cancer. The staff of each center invited patients to participate and those who accepted were sent a link to the questionnaires. The allocation of questionnaires was randomized using the random function of the Excel program. One group received the link to the survey containing the DASS-21, SWLS, FS, and PANAS (*N* = 143; 54 items), whereas the second group received the link to the DASS-21, PSS-10, and SBCS (*N* = 146; 55 items). All participants signed an informed consent form specifying that the information collected was anonymous and confidential and would be used for research purposes only. There were no missing data as the questionnaires could not be submitted electronically unless all items had been responded to.

### Data analysis

First, a descriptive analysis was carried out for each item, calculating the mean, standard deviation, skewness, and kurtosis. Next, in order to obtain validity evidence based on the internal structure of the DASS-21, we conducted a confirmatory factor analysis (CFA) with the* R* package lavaan^58^, testing the structure of three first-order factors and one second-order factor. The method used was diagonally weighted least squares (DWLS) estimation with the polychoric correlation matrix, which has been shown to provide accurate parameter estimates when dealing with categorical items^59^. The chi-square statistic (χ^2^) and the following fit indices were calculated: comparative fit index (CFI), non-normed fit index (NNFI), and the root mean square error of approximation (RMSEA) and its 90% confidence interval. These indexes were interpreted according to the following criteria: CFI and NNFI values of 0.95 or higher are considered to indicate satisfactory fit; RMSEA values between 0.06 and 0.08 indicate a reasonable fit^56^ and those below 0.06 a satisfactory fit.

The reliability (in terms of internal consistency) of DASS-21 scores was then assessed by calculating McDonald’s omega coefficient (ω), considering values of 0.70 or higher as satisfactory. Item analysis was also performed, calculating the corrected item-total correlation coefficient and interpreting values above 0.30 as satisfactory.

Validity evidence based on relationships with other variables was obtained by calculating Pearson correlation coefficients between scores on the three DASS-21 scales (Depression, Anxiety, and Stress), as well as the total score (general psychological distress), and scores on life satisfaction (SWLS), positive and negative affect (PANAS), flourishing (FS), perceived stress (PSS-10), and cancer-specific stressors (SBCS). Coefficients were interpreted in accordance with Cohen's criteria: close to |.10|, low; close to |.30|, moderate; close to |.50| or higher, strong correlation^60^.

Finally, descriptive statistics for scores on DASS-21 scales and its total score were computed. A one-way analysis of variance (ANOVA) was performed to analyze mean differences in scores on Depression, Anxiety, Stress, and total score as a function of time since diagnosis (< 2 years, 2–5 years, and > 5 years) and cancer stage (0-I, II, and III-IV). For the ANOVAs we followed the guidelines proposed by Blanca et al.^61^ regarding heterogeneity of variance and control of Type I error, taking into account the variance ratio, the pairing of variance with group size, and the coefficient of sample size variation.

## Results

### Descriptive analysis for DASS-21 items

Table 2 shows the mean, standard deviation, and skewness and kurtosis statistics for each item. Some skewness and kurtosis values indicate deviation from normality.

**Table 2:** Descriptive statistics for DASS-21 items (N = 289).

Item	M	SD	Skewness	Kurtosis
1	1.61	0.98	− 0.01	− 1.04
2	1.22	1.10	0.37	− 1.19
3	0.93	0.95	0.74	− 0.44
4	0.80	0.98	0.93	− 0.35
5	1.02	0.94	0.56	− 0.65
6	1.22	1.01	0.40	− 0.93
7	0.60	0.95	1.38	0.62
8	1.32	1.05	0.18	− 1.18
9	0.89	0.98	0.73	− 0.66
10	0.83	1.00	0.90	− 0.45
11	1.27	1.04	0.28	− 1.10
12	1.51	1.03	0.04	− 1.15
13	1.47	1.04	0.12	− 1.17
14	0.83	0.82	0.74	− 0.03
15	0.62	0.91	1.30	0.61
16	0.83	0.93	0.81	− 0.39
17	0.65	0.90	1.22	0.46
18	1.18	1.06	0.43	− 1.05
19	1.06	1.03	0.53	− 0.93
20	0.91	1.00	0.80	− 0.50
21	0.61	0.90	1.41	1.00

### Validity evidence based on the internal structure (construct validity)

The CFA results for the second-order factor model showed that all fit indices were satisfactory according to the aforementioned criteria, with values of CFI and NNFI above 0.95 and RMSEA close to 0.06 (Table 3). The standardized parameter values were all significant and are shown in Table 4.

**Table 3:** Fit indices for the second-order factor model of the DASS-21.

Model	χ^2^	df	CFI	NNFI	RMSEA [90% CI]
Three first-order factors and one second-order factor	371.81	186	0.995	0.994	0.059 [0.050–0.068]

N = 289; χ^2^ = chi-square; *df* = degrees of freedom; CFI = comparative fit index; NNFI = non-normed fit index; RMSEA = root mean square error of approximation with 90% confidence interval.

**Table 4:** Standardized factor loadings for the second-order factor model of the DASS-21 and corrected item-total correlation.

Item	Scale	Second-order factor loading	Factor loading	Corrected item-scale correlation	Corrected item-total correlation
3	Depression	0.90	0.81	0.73	0.69
5			0.75	0.58	0.65
10			0.83	0.71	0.68
13			0.92	0.72	0.79
16			0.82	0.75	0.66
17			0.76	0.66	0.60
21			0.82	0.71	0.63
2	Anxiety	0.96	0.55	0.49	0.46
4			0.67	0.57	0.56
7			0.75	0.64	0.60
9			0.76	0.58	0.64
15			0.88	0.69	0.74
19			0.79	0.68	0.68
20			0.79	0.64	0.67
1	Stress	0.94	0.81	0.65	0.64
6			0.80	0.70	0.71
8			0.90	0.80	0.79
11			0.91	0.83	0.80
12			0.89	0.79	0.72
14			0.61	0.49	0.51
18			0.81	0.69	0.71

### Reliability and item analysis

The results showed that the reliability of DASS-21 scale scores and total score was satisfactory, with values of ω equal to 0.90 for Depression, 0.84 for Anxiety, 0.90 for Stress, and 0.95 for the total score. The item analysis yielded values of the corrected item-total correlation above the cut-off of 0.30 (Table 4).

### Validity evidence based on relationships with other variables

In the first subsample, scores on the DASS-21 showed negative and significant correlations with scores on life satisfaction, flourishing, and positive affect, and a positive correlation with scores on negative affect. In the second subsample, scores on the DASS-21 yielded positive correlations with scores on perceived helplessness, physical appearance and sex strains, health and daily difficulties, interpersonal relationship strains, healthcare strains, worries and concerns about the future, and the SBCS total score, and a negative correlation with scores on perceived self-efficacy. These results are shown in Table 5.

**Table 5:** Correlation between scores on the DASS-21 and scores on life satisfaction, flourishing positive and negative affect, perceived helplessness, perceived self-efficacy, and the SBCS subscales.

	*N*	Depression	Anxiety	Stress	Total score
Life satisfaction	143	− 0.49***	− 0.33***	− 0.32***	− 0.41***
Flourishing	143	− 0.58***	− 0.36***	− 0.33***	− 0.46***
Positive affect	143	−0.37***	− 0.24**	− 0.15*	− 0.27***
Negative affect	143	0.46***	0.47***	0.55***	0.54***
Perceived helplessness	146	0.69***	0.65***	0.76***	0.77***
Perceived self-efficacy	146	− 0.24**	− 0.24**	− 0.33***	− 0.30***
Physical appearance and sex strains	146	0.32***	0.32***	0.32***	0.35**
Health and daily difficulties	146	0.34***	0.39***	0.38***	0.40***
Interpersonal relationship strains	146	0.39***	0.35***	0.33***	0.39**
Healthcare strains	146	0.36***	0.39***	0.39***	0.41***
Worries and concerns about the future	146	0.45***	0.45***	0.46***	0.50***
SBCS total score	146	0.45***	0.47***	0.46***	0.50***

*p < .05, **p < .01, ***p < .001.

### Descriptive statistics for DASS-21 scale scores

Table 6 shows the mean and standard deviation for the three DASS-21 scale scores and total score. Considering the cut-off points established for each scale, prevalence was 25.6% for depression (mild 14.6%, moderate 10%, and severe 1%), 33.2% for anxiety (mild 9.7%, moderate 15.6%, severe 6.2%, and extremely severe 1.7%), and 17.6% for stress (mild 11.7%, and moderate 5.9%).

**Table 6:** Descriptive statistics for DASS-21 scale scores in the total sample and as a function of time since diagnosis.

Scale	Total sample		< 2 years		2 – 5 years		> 5 years		
	(*N* = 289)		(*N* = 123)		(*N* = 84)		(*N* = 82)		
	M	SD	M	SD	M	SD	M	SD	F
Depression	6.34	5.22	6.63	5.21	7.18	5.52	5.06	4.73	3.80*
Anxiety	6.11	5.04	6.26	5.12	7.27	5.50	4.70	4.03	5.71**
Stress	8.95	5.57	9.33	5.56	10.11	5.92	7.20	4.80	6.41**
Total score	21.40	14.46	22.22	14.18	24.56	15.75	21.41	14.46	6.31**

*p < .05, **p < .01.

The ANOVA results showed no significant differences according to cancer stage for any scale score. As for time since diagnosis, the test was found to be robust to heterogeneity of variances with the characteristics of the sample data obtained, taking into account the variance ratio, the pairing of variance with group size, and the coefficient of sample size variation^61^. The results revealed differences in means, specifically, mean scores on Depression, Anxiety, Stress, and General psychological distress (total score) were significantly lower in patients with more than 5 years since diagnosis compared with both other sub-groups, whereas there were no differences between those with less than 2 years and those with between 2 and 5 years since diagnosis (Table 6).

## Discussion

The aim of this study was to perform a comprehensive analysis of the psychometric properties of the DASS-21 in a sample of Spanish women with breast cancer, examining validity evidence based on the internal structure, the reliability of test scores and item analysis, and validity evidence based on relationships between DASS-21 scores and life satisfaction, flourishing, positive and negative affect, perceived stress, and stressors in breast cancer. The results extend knowledge about the use of the DASS-21 in the breast cancer context.

Regarding the internal structure, the results provide support for the second-order factor model proposed initially by Henry and Crawford^36^ and corroborated in subsequent studies with different populations^21,30–32,36,40,41^. This suggests that the constructs of depression, anxiety, and stress (measured by the three DASS-21 scales) may be subsumed under the higher-order construct of general psychological distress (reflected in the DASS-21 total score), with higher scores indicating higher levels of distress. These results support the use of the DASS-21 as a screening tool to determine the level of psychological distress among women diagnosed with breast cancer, an important first step in the process of targeting psycho-oncological intervention.

Scores on the DASS-21 also showed satisfactory reliability. Values of ω were 0.90 for depression, 0.84 for anxiety, 0.90 for stress, and 0.95 for general psychological distress, all within the range reported previously^21,25–28,39–46^. Homogeneity indices were also satisfactory for all items.

Regarding validity evidence based on the relationship between DASS-21 scores and well-being indicators, moderate and negative correlations were obtained between scores on all scales of the DASS-21 and those on measures of life satisfaction, flourishing, and positive affect, whereas a positive association was observed with negative affect. This means that patients with high levels of depression (who feel discouraged, blue, and pessimistic about the future), those with high levels of anxiety (who feel apprehensive and worried about a possible loss of control), and those with high levels of stress (who feel overexcited and tense) tend to feel unfulfilled and dissatisfied with their life, are less likely to experience happiness and enthusiasm, and are more prone to negative emotions such as fear, irritability, etc. These results are consistent with previous studies both in the general population^26,28–30,36–40,43^ and with breast cancer patients^5^. With respect to stress-related variables, and in line with previous research^57^, scores on the DASS-21 correlated positively and strongly with scores on perceived helplessness, and moderately and negatively with scores on perceived self-efficacy. These results indicate that patients with high scores on the DASS-21 show a greater tendency to experience their daily life situations as unpredictable and uncontrollable, and are less likely to perceive themselves as able to cope with current stressors.

Considering stress as a stimulus, all DASS-21 scores correlated moderately and positively with scores on the SBCS, a tool designed to measure breast cancer-specific stressors. These results indicate that women with high levels of distress tend to experience stressors in different spheres of the cancer process, specifically those related to body appearance and sex strains, states of physical discomfort such as fatigue or pain, social relationships including family and friends, healthcare strains, and fears about what life may bring in the short or long term. It is worth noting here that the strongest correlations were those between general psychological distress (DASS-21 total score) and worries about the future and the SBCS total score. This is consistent with previous research showing that cancer survivors who experience concerns or fears about the future, often referred to as Damocles syndrome, tend to manifest greater psychological distress^7,17^. These worries about the future in breast cancer patients should therefore be a target of intervention by psycho-oncologists, as they seem to be one of the most important concerns affecting these women. The fact that it was the DASS-21 total score (rather than individual scale scores) which showed the strongest correlation with breast cancer-specific stressors suggests that the instrument should be used in its entirety. That is to say, researchers and clinical psychologists who wish to use the DASS-21 as a screening tool with these patients are advised to administer all three of the instrument's scales (Depression, Anxiety, Stress). Using only part of the instrument^26,42,48^ may yield a less accurate or inconclusive picture of the overall level of psychological distress experienced by a woman with breast cancer.

In relation to the levels of psychological distress found in the present sample, the highest prevalence was for anxiety (33.2%), followed by depression (25.6%), and stress (17.6%), although extreme levels were not very common for any of the three. The results regarding anxiety are similar to those obtained in other studies^4^ in the breast cancer population, which place the percentage of women showing psychological maladjustment (related mainly to anxiety, along with a lower level of optimism and well-being) at 38.24%. The mean scores obtained on the three scales (Depression: 6.34, Anxiety: 6.11, Stress: 8.95; General psychological distress: 21.4) are slightly higher than those reported by Ruiz et al.^25^ in a sample (n = 813 participants) of the general Spanish population, 71% of whom were women aged between 18 and 82 (Depression: 4.42, Anxiety: 3.53, Stress: 6.74; General psychological distress: 14.68). In the present sample, means were lower in patients with more than 5 years since diagnosis, whereas there were no differences between those with less than 2 years and those with between 2 and 5 years since diagnosis. These results suggest that the first five years following diagnosis are key. During this period, patients have to cope with major changes in their lives that drastically disrupt personal, family, social, and work routines. The importance of monitoring anxiety and depression during the first five years after a cancer diagnosis and identifying factors associated with these conditions has been highlighted previously^7,62^.

This study has a number of limitations. First, the questionnaires administered are all self-report instruments and might therefore be affected by response bias. Other tools, such as a diagnostic interview, could have been used to complement the information obtained through self-report. Second, the use of a convenience sample and the characteristics of the sample may limit the generalization of results. Third, the study was cross-sectional and there was no second test administration, so test–retest reliability was not analyzed. Future studies should focus on analyzing reliability in terms of stability of scores. Finally, although the study provides validity evidence with respect to a wide set of variables, the relationships with other potentially important variables such as self-esteem, resilience or quality of life have not been studied.

Despite these limitations, the study provides detailed information about the psychometric properties of a scale that is able to measure general psychological distress in Spanish women diagnosed with breast cancer. In addition to supporting the factor structure and reliability of the original scale, the results show that the DASS-21 is a valid instrument for use in this population. The negative relationship observed between DASS-21 scores and indicators of well-being, and the positive relationship between DASS-21 scores and stress-related variables provides useful information for the design of psychological interventions in the context of breast cancer. This is important because breast cancer is known to be one of the most stressful life events that a woman may face during her lifetime^3,4,7^. Women with high scores on the DASS-21 should be prioritized for psychological intervention to reduce their psychological distress.

## Data Availability

The dataset used are available at 10.24310/riuma.28123.
